# Genital Ulcer of Behçet Disease Localized in the Vagina May Lack Pain, Making It Difficult to Assess

**DOI:** 10.1155/2019/2953676

**Published:** 2019-06-10

**Authors:** Soichiro Obata, Koji Kobayashi, Misaki Toda, Etsuko Miyagi, Shigeru Aoki

**Affiliations:** ^1^Perinatal Center for Maternity and Neonates, Yokohama City University Medical Center, Yokohama, Japan; ^2^Center for Rheumatic Disease, Yokohama City University Medical Center, Yokohama, Japan; ^3^Department of Obstetrics and Gynecology, Yokohama City University Hospital, Yokohama, Japan

## Abstract

Genital ulcer is one of the main clinical symptoms of Behçet disease; ulcers mostly occur in the vulva and are usually quite painful. We present an unusual case of Behçet disease wherein a painless genital ulcer was localized in the vagina. Our case is of a 43-year-old woman diagnosed with Behçet disease that was controlled with prednisolone. She became pregnant and developed fever, oral ulcers, and arthralgia at 16 weeks of gestation. Although a relapse of Behçet disease was suspected, ulceration and pain of the vulva were not observed. At 18 and 28 weeks of gestation, a vaginal ulcer was observed during regular prenatal examination, but the patient had no pain or other symptoms in the vulva. The vaginal ulcer healed at 29 weeks of gestation. No recurrence of the vaginal ulcer and other symptoms of Behçet disease were seen on subsequent follow-up examination at 1 month after delivery. Although genital ulcers of Behçet disease are common in the vulva and are generally painful, if they are located in the vagina, they can be painless. If Behçet disease is suspected based on other symptoms, a vaginal examination should be conducted as necessary for accurate evaluation of Behçet disease.

## 1. Introduction

Behçet disease presents with systemic symptoms triggered by vasculitis [1–3]. Genital ulcer is one of the main clinical symptoms; ulcers mostly occur in the vulva and are usually quite painful [1–3]. We present an unusual case of Behçet disease wherein a painless genital ulcer was localized in the vagina.

## 2. Case Presentation

**Figure 1:**
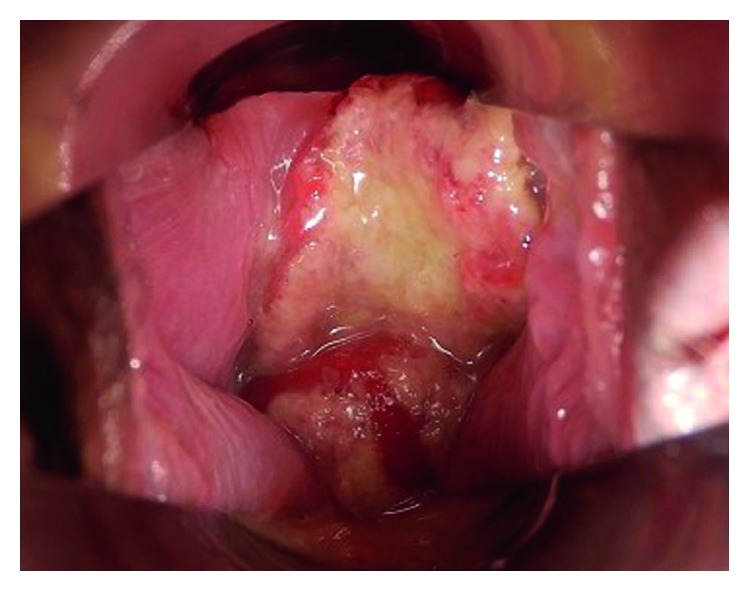
Genital ulcer localized in the vagina at 18 weeks of gestation.

**Figure 2:**
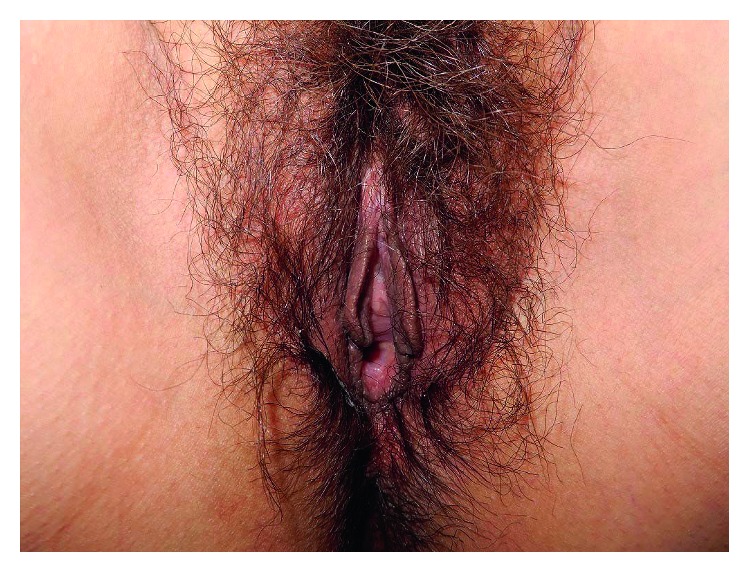
There were no symptoms in the vulva at 18 weeks of gestation.

**Figure 3:**
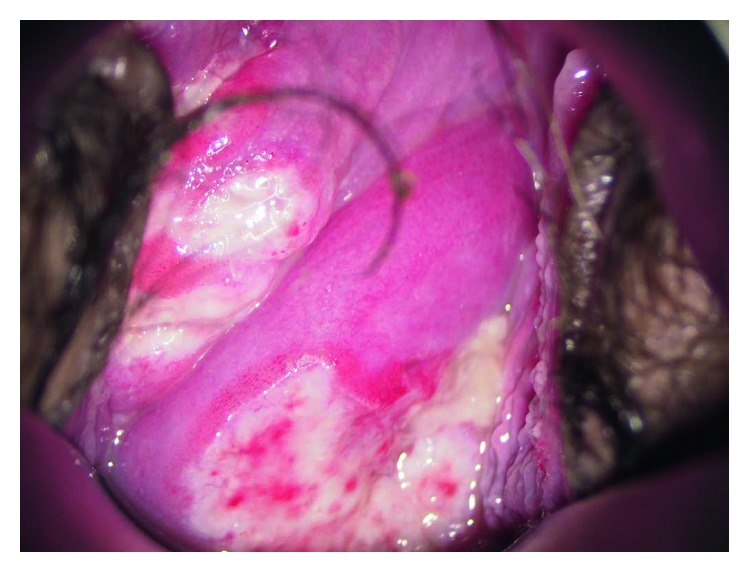
Relapse of vaginal ulcer at 28 weeks of gestation.

**Figure 4:**
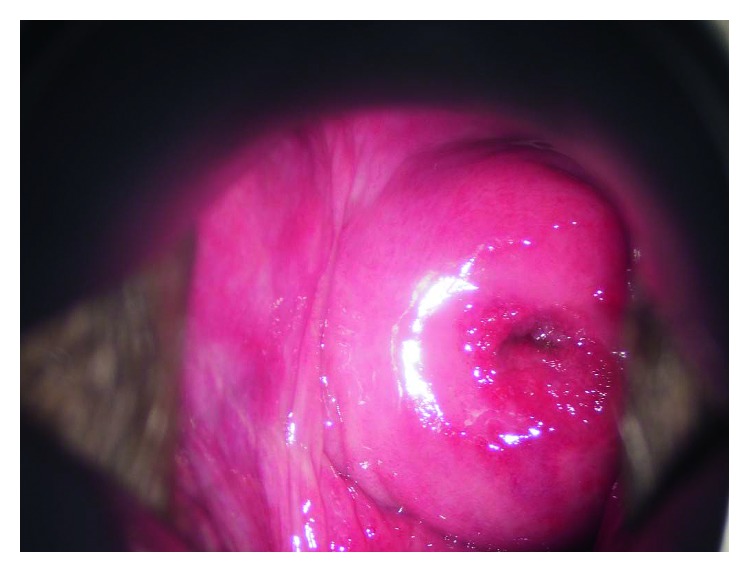
Vaginal ulcer was healed in the postpartum period.

Our case is that of a 43-year-old primiparous woman. She was diagnosed with Behçet disease at the age of 34 years; she presented with recurrent oral ulceration, recurrent genital ulceration, and pseudofolliculitis with characteristic acneiform nodules. She had no eye lesions. Her condition was well controlled with prednisolone (PSL) 8 mg/day oral dose. She fell pregnant spontaneously and visited our perinatal center for her prenatal care. As she was detected with hypertension at her first visit, we recommended that she check her blood pressure regularly at home. She developed fever, oral ulcers, and arthralgia at 16 weeks of gestation, due to which a relapse of Behçet disease was suspected. Because her condition was controlled well with PSL, the daily oral dose of PSL was increased from 8 mg to 10 mg. At this time, ulceration and pain of the vulva were not observed. She visited our outpatient clinic for a regular prenatal examination at 18 weeks and 4 days of gestation, which was when an ulcer localized in the vagina was incidentally observed (Figure 1). The ulcer was painless and the patient had no symptoms in the vulva either (Figure 2). Chlamydial and gonococcal infection were not detected, and cytology of the vaginal wall showed no findings suggesting malignancy. As the other symptoms of Behçet disease were resolved after the dose of PSL had been increased, the same dose was maintained. At the next prenatal care visit, at 22 weeks and 4 days of gestation, the vaginal ulcer had disappeared and other symptoms of Behçet disease were not seen. At 28 weeks and 0 days of gestation, atypical genital bleeding and vaginal ulcer recurrence were observed (Figure 3). At the same time, recurrence of the oral ulcer and arthralgia as well as a mild increase in the fever were observed; based on these symptoms, she was diagnosed with a relapse of Behçet disease and the daily dose of PSL was increased from 10 mg to 15 mg. The vaginal ulcer disappeared at 29 weeks and 2 days of gestation (Figure 4). Thereafter, there was no relapse of the symptoms of Behçet disease.

At 33 weeks and 0 days of gestation, her blood pressure had increased, due to which she was admitted to the hospital. She was diagnosed with severe preeclampsia superimposed. Induction of labor was conducted but it was not effective, and we performed cesarean section at 37 weeks and 6 days of gestation. She delivered a male infant weighing 2002 g. There were no complications in the postoperative course, and she was discharged with her baby at 7 days after cesarean section. No recurrence of the vaginal ulcer and other symptoms of Behçet disease were seen on subsequent follow-up examination at 1 month after delivery.

The patient was explained about the possibility of publishing this case as a case report and the accompanying images. She provided consent.

## 3. Discussion

In Behçet disease, genital ulcers are a common symptom. However, they primarily occur in the vulva and are often painful [1–3]. However, in rare cases, ulcers can be localized in the vagina; in such cases, the ulcers may be painless. Intravaginal examination is usually not performed during regular medical check-ups; in cases with painless vaginal ulcer, there is a possibility of missing the ulcer, resulting in insufficient evaluation of Behçet disease.

In this case, although there were no vulval symptoms, she was diagnosed with a relapse of Behçet disease based on the presence of oral ulcers, arthralgia, and mild increase in fever. In our patient, the painless vaginal ulcer was incidentally noticed during a regular prenatal examination. Senusi et al. examined 137 cases of Behçet disease with primary genital lesions and reported that only 9% patients had ulcers in the vagina; the remaining ulcers were located in the labia or vulva [4]. When a vaginal ulcer is detected, it is necessary to rule out infectious diseases, malignant tumors, and Crohn's disease [5–7]. As the case patient was pregnant, HIV, syphilis, chlamydia, and gonococcal infections had been ruled out due to the regular prenatal examinations. Moreover, cytology of the ulcer yielded no findings indicating malignancy. The patient had no gastrointestinal symptoms that would suggest Crohn's disease. The vaginal ulcer was healed after the PSL dose was increased for relieving the other symptoms of Behçet disease, mainly the intraoral ulcers and arthralgia. Therefore, we diagnosed the vaginal ulcer as one of the symptoms of relapse of Behçet disease.

When ulcers occur in the vulva, such as in Behçet disease or vulvar herpes, it generally causes severe pain [8]. However, most vaginal lesions are painless because there is no nerve ending in the upper part of the vaginal wall. For example, in cases of vaginal wall erosion occurring after pessary use for uterine prolapse, patients report no pain [9]. Similarly, in cases of lacerations limited to the superficial vaginal wall after normal delivery, very few complaints of pain are noted. Moreover, unless there is sexual intercourse, pain may not manifest due to the absence of physical contact. Our patient had not had sexual intercourse as she was pregnant; therefore, pain and bleeding were not observed.

If the genital ulcer is localized in the vagina, the clinical symptoms may be poor and there is a possibility that the evaluation of Behçet disease will be insufficient. Although the other symptoms of Behçet disease were observed in our case, it was initially judged that there was no genital ulcer because there was no ulcer or pain in the vulva. The ulcer was an incidental finding during a regular prenatal examination. In general medical examination, if there is no pain or other symptoms, detailed genital examination is usually not performed, possibly causing insufficient assessment of Behçet disease. In recent years, there is a recommendation for checking the symptoms of intestinal Behçet disease for sufficient evaluation [10]. In a similar vein, accurate genital assessment is also needed. If there is a vaginal ulcer, it is often accompanied by vaginal bleeding. Thus, if relapse of Behçet disease is suspected based on other symptoms, the presence or absence of vaginal bleeding should be confirmed. Especially, in a patient with vaginal bleeding without a vulval ulcer, a vaginal examination should be conducted by a gynecologist for a more accurate evaluation of Behçet disease. Vaginal examination is also needed for ruling out other diseases, such as cervical or endometrial cancer.

To conclude, although genital ulcers of Behçet disease are common in the vulva and are generally painful, if they are located in the vagina, they can be painless. If Behçet disease is suspected based on other symptoms, a vaginal examination should be conducted as it is necessary for an accurate evaluation of Behçet disease, especially in patients with vaginal bleeding without vulval ulcers.
